# Natural Products for the Treatment of Pulmonary Hypertension: Mechanism, Progress, and Future Opportunities

**DOI:** 10.3390/cimb45030152

**Published:** 2023-03-13

**Authors:** Zuomei Zeng, Xinyue Wang, Lidan Cui, Hongjuan Wang, Jian Guo, Yucai Chen

**Affiliations:** 1 School of Traditional Chinese Medicine, Beijing University of Chinese Medicine, Beijing 100029, China; 2 School of Chinese Pharmacy, Beijing University of Chinese Medicine, Beijing 100029, China

**Keywords:** pulmonary hypertension, natural products, pharmacology, plant extracts, pulmonary vascular remodeling

## Abstract

Pulmonary hypertension (PH) is a lethal disease due to the remodeling of pulmonary vessels. Its pathophysiological characteristics include increased pulmonary arterial pressure and pulmonary vascular resistance, leading to right heart failure and death. The pathological mechanism of PH is complex and includes inflammation, oxidative stress, vasoconstriction/diastolic imbalance, genetic factors, and ion channel abnormalities. Currently, many clinical drugs for the treatment of PH mainly play their role by relaxing pulmonary arteries, and the treatment effect is limited. Recent studies have shown that various natural products have unique therapeutic advantages for PH with complex pathological mechanisms owing to their multitarget characteristics and low toxicity. This review summarizes the main natural products and their pharmacological mechanisms in PH treatment to provide a useful reference for future research and development of new anti-PH drugs and their mechanisms.

## 1. Introduction

Pulmonary hypertension (PH) is a severe disease characterized by the involvement of small distal pulmonary vessels, pulmonary vasoconstriction, functional dysfunction of pulmonary artery endothelial cells (PAECs), pulmonary arterial smooth muscle cells (PASMCs) proliferation, and in situ thrombosis. These pathogenetic factors result in a progressive increase in pulmonary vascular resistance, pulmonary arterial pressure (PAP), and restricted blood flow, leading to impaired right heart function and death [1]. The hemodynamic diagnostic criteria are defined as a mean PAP (mPAP) ≥ 25 mmHg measured by supine resting right heart catheterization [2]. Epidemiology has shown that the clinical prognosis of patients with PH is poor. Without treatment, many patients with PH and right ventricular failure die [3]. Accurate diagnosis and pharmacological treatment are the keys to improving patient survival. However, PH is a multifactorial clinical pathophysiological syndrome with complex pathological mechanisms that need to be explored [4].

The main drugs currently used to treat PH include endothelin receptor antagonists, prostacyclin and its analogs, and phosphodiesterase-5 inhibitors [5]. They mostly exert their therapeutic effect by regulating the imbalance between vasoconstriction and vasodilation. In patients with PH, endothelin-1 (ET-1) is often overexpressed, causing vasoconstriction and PASMC proliferation. Bosentan, a representative endothelin receptor antagonist, antagonizes ETA and ETB receptors to achieve vasodilatory and antiproliferative effects [6]. Prostacyclin can increase cAMP concentration in smooth muscle cells and dilate blood vessels. It also antagonizes the endothelin effect and inhibits platelet activation, vascular remodeling, and in situ thrombosis [7]. In the pathological progression of PH, nitric oxide (NO) production is reduced, and vasoconstriction occurs. Sildenafil, a phosphodiesterase-5 inhibitor, increases endogenous NO levels and inhibits platelet activation and vascular remodeling [8]. These drugs improve patient prognosis moderately, but their clinical application is limited. Bosentan causes liver damage, and sildenafil has age restrictions [9,10]. In addition, these drugs are simply designed from a single target, which limits their clinical applications. Therefore, there is an urgent need to develop a multitarget, safe, and low-cost treatment for PH.

**Figure 1:**
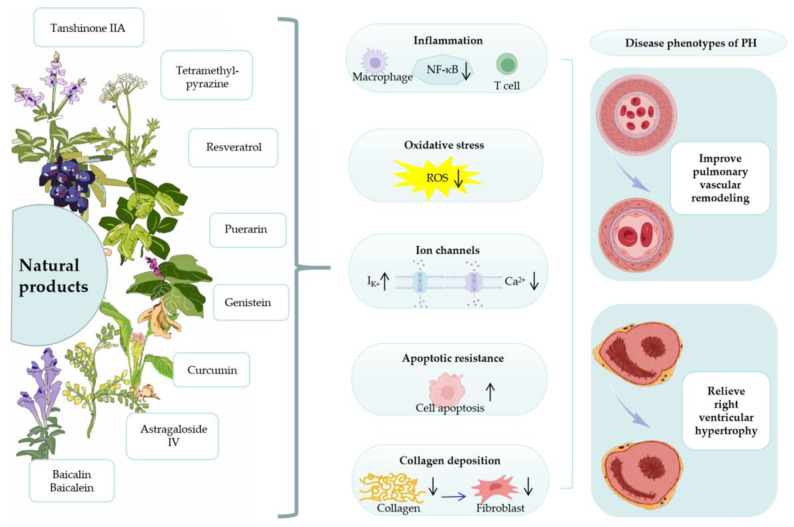
Some deeply studied natural products can play a role in the treatment of pulmonary hypertension by improving inflammatory responses, inhibiting oxidative stress, reducing apoptotic resistance, and regulating abnormal ion channels and collagen deposition, which can effectively improve pulmonary vascular remodeling and right ventricular hypertrophy. Abbreviations: PH, pulmonary hypertension; NF-κB, nuclear factor-κB; and ROS, reactive oxygen species.

We focused on natural products, which have been shown to have unique advantages in treating PH. This is because they have a multitarget and multipathway synergistic effect in treating the disease, with significant efficacy in inhibiting pulmonary vascular remodeling (PVR) and protecting right heart function [11,12,13]. The specific mechanism may be closely related to improving inflammatory response, inhibiting oxidative stress, reducing cell apoptosis resistance, and regulating abnormal ion channels and collagen deposition [14,15,16,17,18]. In addition, most natural products are extracted from purely natural plants, therefore the drugs meet the requirements of safety and low cost. It is reasonable to speculate that natural products for PH treatment have great potential for development. This paper reviewed the main natural products for PH treatment and their pharmacological mechanisms (Figure 1). We also explored potential PH therapeutic mechanisms to provide a useful reference for future research and development of new anti-PH drugs and their mechanisms.

## 2. Pathological Mechanisms of PH

The pathogenesis of PH includes pulmonary vasoconstriction, PVR, and thrombosis in situ, resulting in small and medium-sized pulmonary artery occlusion, which can eventually lead to right ventricular hypertrophy (RVH) or even failure [19]. Pulmonary vasoconstriction can directly lead to increased pulmonary vascular resistance, which is one of the causes of PH. The disruption of homeostasis between vasoconstrictive and diastolic substances can result in pulmonary vasoconstriction [20]. In addition, pulmonary vasoconstriction has a complex relationship with the activity of ion channels and the molecular mechanisms associated with hypoxia [21]. PVR is a key structural change in PH. It refers to the proliferation of PAECs, PASMCs, fibroblasts, and other structural cells in pulmonary blood vessels, accompanied by abnormal accumulation of extracellular matrices such as collagen fibers and elastin. This process can cause thickening of the intima and media of pulmonary vessels, resulting in lumen stenosis and irreversible PH progression [22]. Multiple molecular mechanisms, such as persistent inflammation and immune disorders, dysregulation of proliferative and apoptotic signaling pathways, endothelial-to-mesenchymal transition (EndMT), and genetic alterations, induce PVR [23,24]. Endothelial dysfunction, inflammation, platelet activation, and fibrinolysis are risk factors for thrombosis in pulmonary vessels [25,26].

In general, the pathogenesis of PH is complex, and it is difficult to achieve satisfactory therapeutic effect only by targeting a single pathological mechanism. All of the above mechanisms are indispensable factors leading to the occurrence and development of PH. Existing targeted drugs are available to effectively address pulmonary vasoconstriction, but there are no definitive drugs that effectively mitigate PVR, in situ thrombosis, and progressive hypertrophy of the right ventricle [27]. In recent years, several studies have shown that natural products can simultaneously intervene in multiple parts of the disease because of multitargets and multipathways. They have significant advantages in the treatment of complex diseases such as PH [28]. Therefore, in the present review, we reviewed the most popular natural products for treating PH and classified the mechanisms involved in these natural products.

## 3. Natural Products for the Treatment of PH

### 3.1. Tanshinone IIA

Tanshinone IIA (TIIA) is the main active component of traditional Chinese medicine (TCM), *Salvia miltiorrhiza* Bunge, which can improve PH by restoring ion channels, reducing inflammation, and counteracting apoptosis resistance [29,30]. Chronic exposure to hypoxia decreased the mRNA and protein expression of KV1.5 and KV2.1 in small pulmonary artery PASMCs in rats and led to an increase in basal intracellular Ca^2+^ concentration ([Ca^2+^]_i_) and store-operated Ca^2+^ entry (SOCE) [31,32]. TIIA modulates KV1.5 and KV2.1 and upregulates the expression of IKV currents in small pulmonary arteries [33]. Sodium tanshinone IIA sulfonate (STS), a water-soluble salt of TIIA, reduces SOCE and basal calcium ions in PASMCs of chronic hypoxic pulmonary hypertension (CHPH) rats by inhibiting the expression of canonical transient receptor potential (TRPC) 1 and TRPC6, thereby reducing pulmonary vascular resistance [34]. In addition, STS can prevent the hypoxia-mediated increase in intracellular calcium homeostasis and cell proliferation by targeting the hypoxia-inhibited protein kinase G-peroxisome proliferator-activated receptor-γ (PKG-PPAR-γ) signaling pathway in PASMCs [35].

STS increased the protein stability of the bone morphogenetic protein type 2 receptor (BMPR2) and inhibited lysosomal degradation of the BMPR2 protein, enhancing bone morphogenetic protein 9 (BMP9)-BMPR2-small mothers against decapentaplegic homolog (Smad1/5/9) signaling transduction in PMVECs, thereby significantly inhibiting hypoxia-induced apoptosis in the lung endothelium and primary cultured PMVECs of HPH rats [36]. The phosphoinositide-3-kinase/protein kinase B/mammalian target of rapamycin (PI3K/Akt/mTOR) pathway plays an important role in inducing PVR and fibrosis [37]. STS dose-dependently promotes apoptosis of PASMCs by inhibiting the PI3K/AKT/mTOR pathway and reduces PH pulmonary edema by decreasing the inflammatory response [38].

As an effective treatment for PH, the efficacy and safety of STStreatment for PH has been placed in Phase 3 clinical trials. In clinical trials of STS for PH, STS significantly improved patients’ exercise capacity and dyspnoea index, and the WHO functional class was reduced from III or IV to II, with high clinical efficacy [39].

### 3.2. Other Active Natural Products Derived from Danshen

Salvianolic acid A (SAA), magnesium lithospermate B (MLB), and Danshensu are all bioactive components from *Salvia miltiorrhiza* Bunge, which can improve PH by inhibiting oxidative stress, reducing inflammation, and reversing EndMT. EndMT is the process by which endothelial cells lead to a shift towards mesenchymal cellular phenotypes and functional responses under various stimuli such as hypoxia and inflammation. Several studies have shown that EndMT is involved in PVR [24,40]. SAA protects human pulmonary arterial endothelial cells (HPAECs) and alleviates hypoxia-induced EndMT through resistance to oxidative stress [41]. In addition, SAA can inhibit transforming growth factor-β1 (TGFβ1)-induced EndMT by activating the NF-E2-related factor 2/heme oxygenase-1 (Nrf2/HO-1) pathway and reducing intracellular reactive oxygen species (ROS) production, thus inhibiting PVR [42].

MLB has a preventive effect on PH by blocking phenotypic transformation of pulmonary arteries in hypoxic PH rats, an effect achieved by inhibiting the nicotinamide adenine dinucleotide phosphate (NADPH) oxidases (NOX)/ROS/extracellular signal-regulated kinase (ERK) pathway [43]. In addition, MLB can reduce NOX and vascular peroxidase-1 (VPO1) protein levels by inhibiting the NOX/VPO1 pathway and improving RVH in rats with PH [44]. MLB also inhibits hypoxia-induced EndMT and reverses hypoxia-induced elevation of hypoxia-inducible factor-1α (HIF-1α), monocyte chemoattractant protein-1 (MCP-1), nuclear factor-kappa B (NF-κB), proliferating cell nuclear antigen (PCNA), CDK4, and cyclinD1 mRNA expression in the lungs of PH rats [45]. Danshensu inhibits hypoxia-induced proliferation of PASMCs through a TGF-β-smad3-related pathway [46]. Extract of *Salvia przewalskii* can repair tissue damage from chronic hypoxia by down-regulating the expression of HIF-1α, PCNA, B-cell lymphoma-2 (Bcl-2), and other cell proliferation-related cells, as well as by inhibiting pro-inflammatory cytokines (MCP-1 and NF-κB) and the RhoA–Rho-associated protein kinase (ROCK) signaling pathway [47].

### 3.3. Tetramethylpyrazine

Tetramethylpyrazine (TMP), also known as ligustrazine, is a compound isolated from the TCM *Rhizoma Chuanxiong*. TMP is used to treat cardiovascular diseases based on its anti-inflammatory and anticoagulation effects [48]. It is an effective and safe treatment for PH [49]. TMP inhibits proliferation of PASMCs by blocking G0/G1 to S-phase progression through the PI3K/Akt signaling pathway, thereby attenuating monocrotaline (MCT)-induced PH in rats [50]. Hypoxia stimulates the activation of calcium-sensing receptors (CaSRs) in PASMCs to increase PAP, and TMP can reduce the mRNA and protein levels of CaSRs to treat PH [51]. Vascular leakage due to endothelial barrier dysfunction plays an important role in many lung diseases. Hypoxia increases permeability and pulmonary vascular leakage in the PMVECs of rats with PH. In vivo and in vitro studies of TMP for the treatment of PH have shown that TMP reduces pulmonary vascular endothelial leakage in a dose-dependent manner by inhibiting the ROS-HIF-1α-vascular endothelial growth factor (VEGF) pathway [52]. TMP also significantly reduced pulmonary artery pressure in MCT-PH via the ROS/inducible nitric oxide synthase (iNOS)/cGMP-dependent protein kinase 1 (PKG-1) axis, improving pulmonary artery muscularization and RVH [53].

TMP, as a Rho/ROCK inhibitor and Nrf2/antioxidant response element (ARE) activator, can effectively alleviate PH. Recent studies have reported TMP for targeted pulmonary delivery as inhalation aerosols in PH. In vivo results have shown that inhaling from a TMP aerosol, either as an inhaled liquid or as a dry powder, effectively alleviates PH [54]. The efficacy and safety of TMP in the treatment of PH has been put into clinical trials, and it is considered one of the most beneficial drugs in the treatment of PH. In earlier clinical studies of TMP, it was shown that it could reduce mPVP in patients with PH and improve right heart function by dilating the pulmonary vasculature and reducing pulmonary vascular resistance [55].

### 3.4. Resveratrol

Resveratrol, present in grapes, red wine, and peanuts, has anti-inflammatory, antioxidant, and cardioprotective properties [56]. Resveratrol is effective in the treatment of cardiovascular diseases. Many studies have shown a remarkable ameliorative effect of resveratrol in PH through multiple signaling pathways [57]. In a network pharmacological study of resveratrol for PH, it was found that resveratrol could treat PH through multiple biological pathways, such as regulating NO metabolism, inflammatory responses, and smooth muscle cell proliferation [58]. Other studies have shown that resveratrol decreased the expression of inflammatory cytokines (tumor necrosis factor-α, interleukin-6, and interleukin-1 β) in pulmonary arteries, increased the expression of endothelial nitric oxide synthase (eNOS), reduced oxidative stress, and improved the endothelial function of small pulmonary arteries [59]. Sphingosine kinase 1/sphingosine-1-phosphate (SphK1/S1P) signaling induces PVR through the activation of NF-κB and the up-regulation of cyclin D1 expression, and resveratrol reverses this process by inhibiting the SphK1/S1P/NF-κB/cyclinD1 signaling pathway [60]. Resveratrol reduces PASMC proliferation, migration, and RVH by blocking the PI3K/Akt signaling pathway and inhibiting arginase II mRNA expression and arginase activity [61].

Resveratrol has a cardioprotective effect. Resveratrol partially protects mitochondrial integrity by deacetylating cyclosporin-D, increasing sirtuin-3 (SIRT3) expression, and preventing the opening of the mitochondrial permeability transition pore (mPTP). The further rescue of cardiomyocytes is achieved by maintaining sarco–endoplasmic reticulum Ca^2+^-ATPase (SERCA) activity [62]. Silence information regulator 1 (SIRT1) was significantly reduced in the platelet-derived growth factor BB (PDGF-BB)-treated human PASMCs and MCT-induced PH rats. Resveratrol reduces pulmonary artery pressure and pulmonary artery remodeling by upregulating SIRT1 and p21 expression and downregulating cell cycle protein D1 expression [63]. Resveratrol, an activator of the SIRT1 pathway, also affects the antiproliferative phenotype of PASMCs by modulating the ubiquitin–proteasome system. Resveratrol normalizes pulmonary vascular atrogin-1 mRNA expression via the SIRT1 pathway, reverses PH intrapulmonary artery medial thickening, reduces right ventricular systolic pressure, and RVH [64]. Serum and lung metabolomics revealed that resveratrol can treat PH through three key metabolites: hydroxyphenyllactic acid (lung), isopalmitic acid (serum), and cytosine (lung). In addition, resveratrol can also play a therapeutic role in PH through amino acids, tricarboxylic acid (TCA) cycle, choline, and other pathways [65].

### 3.5. Baicalin and Baicalein

Baicalin and baicalein are flavonoids with a wide range of pharmacological activities isolated from the TCM *Scutellaria baicalensis* Georgi. Studies have shown that baicalin and baicalein can play a role in the treatment of PH in many aspects, such as anti-inflammatory, inhibition of PASMC proliferation and EndMT, reduction of oxidative stress, and stabilization of extracellular matrix [66]. The improvement of baicalin on PVR may be achieved through the following pathways alone or together, including the regulating tumor necrosis factor-α (TNF-α)/BMPR2 signaling pathway, downregulating p38 mitogen-activated protein kinase (MAPK)/matrix metalloproteinase-9 (MMP-9) signaling pathway, promoting Akt/eNOS, and inhibiting ERK and the NF-κB signaling pathway [67,68,69]. Baicalein protects against clinical HPH, in part, through enhanced A2A receptor (A2AR) activity and down-regulation of stromal cell-derived factor-1 (SDF-1)/C-X-C chemokine receptor type 4 (CXCR4)-induced PI3K/Akt signaling [70].

Baicalein can significantly reduce right ventricular systolic pressure (RVSP) and improve RVH and PVR in rats with PH. The therapeutic mechanism may be related to Akt/ERK1/2/glycogen synthase kinase-3β (GSK3β)/β-catenin/ET-1/ETAR signaling pathway inhibition and endothelial dysfunction prevention [71]. Baicalein partially inhibited the MAPK and NF-κB signaling pathways, reduced the levels of apoptosis and inflammatory biomarkers in the lung tissue, and improved PVR [72]. In addition, baicalein also partially inhibited pulmonary artery EndMT by modulating the NF-κB-BMPR2 pathway [73]. Recent studies have shown that baicalein reversed MCT-induced PH by targeting the lung–pulmonary arteries (Pas)–PASMCs axis, activating the BCL-2-associated X (Bax)/Bcl-2/caspase-3 (Cas-3) signaling and downregulating the pro-inflammatory cytokine TNF-α, which significantly inhibited PAs and right ventricular remodeling [74].

### 3.6. Puerarin

Puerarin is a natural flavonoid with various pharmacological activities extracted from the herb *Pueraria lobata* (*Willd.*) *Ohwi.* It can be used to treat various cardiovascular diseases [75]. Puerarin ameliorates hypoxia-induced PASMC proliferation in an autophagy-dependent manner by reducing the expression of autophagic markers in vitro and in vivo [76]. Puerarin protects HPAECs from hypoxia-induced apoptosis and improves their viability. Further studies showed that Puerarin could exert a protective effect in both MCT and hypoxic experimental PH rodent models. The mechanism may be closely related to the inhibition of oxidative stress and activation of the BMPRII/Smad and peroxisome proliferator-activated receptor γ (PPARγ)/PI3K/Akt signaling pathways [77]. A novel crystal type V (Puer-V) was found to have a better therapeutic effect than the crude form of puerarin, effectively alleviating abnormal structural changes and dysfunction in lung tissue and the right ventricle [78]. Puerarin also exhibited anti-inflammatory properties in rats with acute lung injury by modulating the renin–angiotensin system and NF-κB signaling pathway, modulating the nucleotide-binding oligomerization domain-like receptor family pyrin domain-containing-3 (NLRP3) inflammasome-induced pyroptosis, and reducing TNF-α, interleukin-6 (IL-6), and interleukin-1β (IL-1β) production [79,80,81].

### 3.7. Genistein

Genistein, an isoflavone extracted from soybeans, puerarin, and other legumes, is a natural phytoestrogen with anti-inflammatory, antioxidant, and cardioprotective effects [82,83]. Network pharmacological studies suggest that genistein may exert PH effects through NO synthesis, apoptosis, and the PPARγ signaling pathway [84]. The hypertrophy of PASMCs is involved in the development of medial pulmonary artery thickening. Genistein may inhibit hypoxia-induced hypertrophy of PASMCs through the estrogen and β-adrenergic receptor signaling pathways [85]. Genistein also attenuates PH in broiler chickens by restoring endothelial function. Through the PI3K/Akt-dependent signaling pathway, genistein stimulates a rapid phosphorylation of eNOS at the Ser1179 site, which is associated with eNOS/NO axis activation, prompting eNOS activation in broiler pulmonary artery endothelial cells [86].

### 3.8. Astragaloside IV

Astragaloside IV is a small purified saponin isolated from the *Astragalus membranaceus* (Fisch.) Bunge. It possesses a wide range of pharmacological properties, including anti-inflammatory, antioxidant, and antiproliferative [87]. Astragaloside IV can alleviate PH by reducing inflammation and PASMC proliferation in vivo and in vitro experiments [88]. In HPAECs, astragaloside IV normalizes hypoxia-induced inflammatory cytokine release, HIF-1α levels, and VEGF [89]. Astragaloside IV also attenuates inflammatory responses mediated by NLRP-3/calpain-1 in the development of PH [90]. Astragaloside IV ameliorates PVR in hypoxia-induced PH by restraining the T follicular helper cell response, expanding the T follicular regulatory cell response, and regulating the Notch signaling pathway [91,92]. In addition, astragaloside IV has been shown to have a protective effect on the heart. It regulates Ca^2+^ homeostasis to inhibit apoptosis and prevent myocardial injury in rats with chronic intermittent hypoxia [93].

### 3.9. Curcumin

Curcumin is a kind of fat-soluble phenolic pigment extracted from the dried rhizomes of *Curcuma longa* L. (*turmeric*) [94]. A recent study showed that curcumin provides vascular protection against arterial hypertension and PH. It can also improve hypertension and vascular remodeling by suppressing vasoconstriction, inhibiting vascular smooth muscle cell proliferation and migration, and improving endothelial cell dysfunction [95]. Curcumin protects mitochondrial function and promotes PASMCs apoptosis, thereby effectively reducing PAP and reversing pulmonary artery remodeling [96]. Curcumin is also used as a nutritional supplement. It can actively improve vascular changes, right ventricular failure, and other complications of PH [97]. Curcumin analogues inhibit phosphodiesterase-5 activity and have a concentration-dependent vasodilatory effect on pulmonary arteries [98]. Curcumin may reduce PH-induced cardiac remodeling by reducing TNF-α levels and oxidative stress [99]. More interestingly, curcumin remarkably alleviated the psychological state of PH patients, and the scores of the self-rating depression scale (SDS) and self-rating anxiety scale (SAS) were lower than the control group [100].

**Figure 2:**
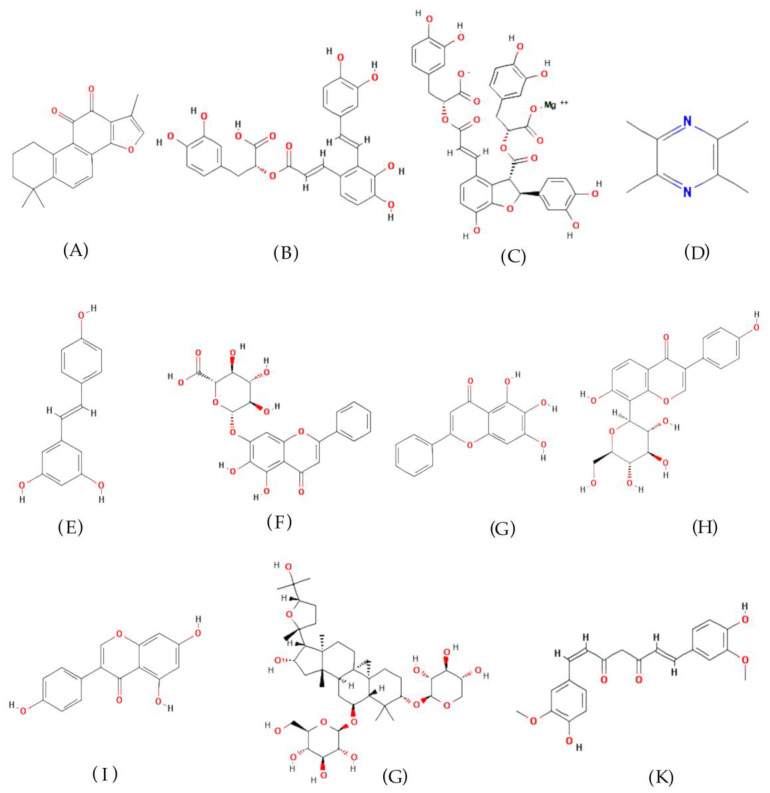
Chemical structures of natural products for the treatment of pulmonary hypertension. (**A**) Tanshinone IIA; (**B**) salvianolic acid A; (**C**) magnesium lithospermate B; (**D**) tetramethylpyrazine; (**E**) resveratrol; (**F**) baicalin; (**G**) baicalein; (**H**) puerarin; (**I**) genistein; (**J**) astragaloside IV; and (**K**) curcumin.

In this section, we present a list of the most popular natural products used to treat PH. Natural products that are more studied and have more therapeutic pathways for PH are ranked first to highlight the natural products with the most potential for the treatment of PH. The chemical structures of these natural products are summarized in Figure 2.

## 4. Mechanism of Natural Products for the Treatment of PH

### 4.1. Anti-Inflammatory

High levels of cytokines, chemokines, and inflammatory mediators were detected in patients with PH, in addition to inflammatory cell infiltration in and around vascular lesions, including T cells, B cells, macrophages, dendritic cells, and mast cells, which characteristically accumulate around pulmonary blood vessels in patients with PH [101,102]. There is substantial evidence that alterations in immune and vascular cells are directly involved in disease progression, promoting PVR and leading to irreversible development of PH, which correlates with disease severity and survival [103]. Inflammation has long been thought to play a crucial role in the development of PH and is a potential therapeutic target for PH [104].

NF-κB is a transcription factor that plays a key role in cell differentiation, survival, and proliferation and regulates inflammatory signaling [105]. The activation of NF-κB exacerbates PH progression, and the inhibition of NF-κB reduces pulmonary artery occlusion [106]. A variety of natural products, such as TIIA, MLB, resveratrol, puerarin, betaine, baicalin, and baicalein, can treat PH by inhibiting NF-κB-related pathways [45,60,69,72,79,107,108,109]. In addition, NF-κB activation has been shown to increase MCP-1 expression and promote PASMCs proliferation. MCP-1 is a member of the chemokine C-C or subfamily that induces monocyte recruitment in acute pulmonary vascular injury, thereby promoting inflammation [110]. Resveratrol reduces monocyte recruitment and pulmonary artery endothelial cell injury by downregulating MCP-1 expression [111,112].

PVR is often accompanied by an increased expression of inflammatory factors such as TNF-α, IL-6, and IL-1β [113]. Resveratrol, puerarin, allicin, grape seed proanthocyanidin, and many other natural products can inhibit lung inflammation by reducing the expression of inflammatory factors such as TNF-α, IL-6, and IL-1β in the lung, thereby reducing pulmonary vasoconstriction and vascular remodeling [59,79,114,115]. Notably, some studies have indicated that IL-6 is one of the most important inflammatory cytokines in the development of PH [116,117]. The IL6-STAT3-miR-17/92-BMPR2 pathway is a potentially valuable mechanism for inhibiting pulmonary artery remodeling [118]. In addition, clinical and preclinical evidence suggests that inflammasomes, particularly NLRP3 and its downstream cytokine products, are promising drug targets for PH [119]. Puerarin inhibits NLRP3 inflammasome-induced pyroptosis to improve acute lung injury [81]. Astragaloside IV has been shown to inhibit MCT-induced PH via the NLRP-3/calpain-1 pathway [90].

### 4.2. Oxidative Stress

When the body is exposed to various harmful stimuli such as hypoxia and smoke, the production of ROS in the lungs is excessive, and oxidation exceeds the scavenging of oxidants, leading to inflammatory infiltration and the increased secretion of proteases [120]. Oxidative/antioxidant imbalances were found in the systemic circulation of PH patients, and elevated pulmonary arterial pressure was associated with increased oxidative stress in three different PH animal models (hypoxia, MCT, and pulmonary embolism-induced) [121,122,123,124]. Multiple studies have shown that ROS is involved in the development and progression of PH, causes pulmonary microvascular endothelial dysfunction, and is also associated with promoting PH-induced RVH and right heart failure [125,126]. With the study of ROS and its signal transduction pathway and the pathogenesis of PH, antioxidant therapy has become a new idea in the prevention and treatment of PH, and many natural products can be used to prevent PH through antioxidative stress.

NADPH oxidase is considered to be the main source of ROS. It has been confirmed that NADPH oxidase can induce PVR either alone or through the production of ROS [127,128]. NADPH oxidase and its downstream components in the ROS signaling pathway may be potential targets for the treatment of PH. MLB and 18β-glycyrrhetinic acid downregulate NOX2 and NOX4 levels, and inhibit hypoxia and MCT-induced oxidative stress [43,129]. NOX acts synergistically with VPO1 to amplify the role of NOX-derived ROS in oxidative damage in PH [130]. MLB and trimethoxystilbene, a novel resveratrol analog, prevented PVR and RVH in rats by inhibiting NOX/VPO1 pathway-mediated oxidative stress and inflammatory responses as well as ERK signaling pathways [44,131]. Wogonin is the active ingredient of *Scutellaria baicalensis* Georgi. Wogonin inhibits the proliferation of PASMCs by modulating the HIF-1/NOX4 pathway [132].

Nrf2 is a major regulator of antioxidant response, which can inhibit oxidative stress by regulating a series of antioxidant expression enzymes, including HO-1 [133]. SAA can inhibit TGFβ1-induced EndMT by activating the Nrf2/HO-1 pathway and reducing intracellular ROS production, thus inhibiting PVR [42]. Resveratrol increased Nrf-2/Thioredoxin 1 (Trx-1) axis expression, inhibited HIF-1α expression, and reduced hypoxia-induced ROS production in PAMSCs by inhibiting the MAPK/ERK1 and PI3K/Akt pathways [134]. In addition, tetramethylpyrazine exerts anti-inflammatory, antioxidant, and antiproliferative effects in pulmonary arteries by inhibiting the ROS-HIF-VEGF pathway and regulating the ROS/iNOS/PKG axis [52,53].

### 4.3. Ion Channels

Dysregulation of ion channels in PH has been widely described, most notably involving calcium channels and potassium channels [135]. Elevated intracellular free calcium ion concentration ([Ca^2+^]_i_) is a key mechanism leading to contraction and remodeling of PASMCs [136]. When PAECs are stimulated by hypoxia and inflammation, a variety of vasoactive factors (such as ET-1 and thromboxane) will be released, resulting in impaired K channel activity in PASMCs. The inhibition or closure of the K channels in PASMCs leads to membrane depolarization, which activates voltage-gated Ca^2+^ channels, leading to increased intracellular Ca^2+^ concentrations and vasoconstriction [137]. Reducing intracellular free calcium ion concentrations and restoring K-channel activity are extremely attractive therapeutic strategies in PH, and natural products mostly exert their therapeutic effects on PH by modulating these two ion channels.

TIIA can produce a dilating effect on pulmonary arteries by inhibiting the inward flow of extracellular Ca^2+^, in part by inhibiting the release of intracellular Ca^2+^ and the activation of K-channels [138]. TRPC channels have been shown to help regulate [Ca^2+^]_i_, either directly by supporting Ca^2+^ influx through the plasma membrane or indirectly by regulating the resting membrane potential [135]. Increased mRNA and protein levels of the typicalTRPC1 and TRPC6 in distal pulmonary artery smooth muscle under hypoxic conditions, in turn, lead to enhanced SOCE. STS can reduce SOCE and basal calcium ions in PASMCs by inhibiting the increase of TRPC1 and TRPC6 in CHPH rats, thereby alleviating pulmonary vascular resistance and improving pulmonary vascular and right ventricular remodeling [34]. CaSRs play an important role in changes in calcium concentrations in PASMCs, pulmonary vasoconstriction, and proliferation. Tetramethylpyrazine significantly inhibits the activation of CaSRs in PASMC in a dose-dependent manner and reduces intracellular calcium ion concentrations [51].

Intermittent and sustained hypoxia decreases mRNA and protein expression of KV1.5 and KV2.1 in small pulmonary arteries and significantly reduces IKV currents in PASMCs [139]. STS significantly inhibits hypoxia-induced proliferation of PASMCs by affecting the expression of Kv2.1 in PASMCs [140]. Extract of the wild orchid, *Eulophia macrobulbon* (EM) reduces extracellular Ca^2+^, inhibits intracellular Ca^2+^ release activated by phenylephrine, and inhibits phosphodiesterase5 (PDE5), thus better dilating pulmonary blood vessels [141]. The active fraction of *Rhodiola tangutica* (Maxim.) S.H. Fu pretreatment alleviated the inhibition of IK, upregulated K^+^ channel protein expression, and inhibited Ca^2+^ channel protein expression, inhibiting hypoxia-induced PASMC proliferation in rats with PH [142].

### 4.4. Apoptotic Resistance

The imbalance between cell death and proliferation occurs at every stage of PH and involves every cell type in the pulmonary vascular system, including but not limited to endothelial cells (ECs), smooth muscle cells (SMCs), and fibroblasts [143]. The presence of highly hyperproliferative and apoptosis-resistant PASMCs is a marker of PVR. Evidence suggests that improving vascular remodeling by inducing apoptosis in PASMCs is an effective strategy for treating PH [144]. Hypoxia significantly reduced the levels of the pro-apoptotic proteins Bax, cleaved caspase-3, and cleaved caspase-9, while it increased the expression of the antiapoptotic protein Bcl-2 in HPASMCs. Astragaloside IV, puerarin, baicalein, and many other natural products can inhibit apoptotic resistance in PVSMCs by modulating apoptotic proteins [74,89,145]. Mitochondria balance cell apoptosis by regulating a family of apoptotic proteins [146]. Apigenin is a widely distributed natural dietary flavonoid that can induce mitochondrial-dependent apoptosis in PASMCs by inhibiting the HIF-1α-KV1.5 channel pathway [147]. Salidroside reverses hypoxia-induced apoptotic resistance in PASMCs via an A2aR-related mitochondria dependent pathway [148]. In addition, salidroside-inhibited hypoxia induced PASMC proliferation via the adenosine monophosphate-activated protein kinase (AMPK)α1-P53 P21/P27 axis, reversed hypoxia-induced apoptosis resistance via the AMPKα1-p53 Bax/Bcl-2 axis, and rebalanced proliferation and apoptosis [149].

### 4.5. Collagen Deposition

Collagen synthesis and accumulation play an important role in PH progression. In PH-lesioned pulmonary arteries, the tissue inhibitor of metalloproteinase (TIMP) is upregulated and inhibits MMP. Their balance is disrupted, promoting extracellular matrix (ECM) deposition and vascular remodeling [18,150]. Baicalin can improve pulmonary arteriolar remodeling by inhibiting the p38 MAPK signaling pathway and the expression of MMP-9, increasing disintegrins and metalloprotease with a thrombospondin type-1 motif (ADAMTS-1) and inhibiting the synthesis of type I collagen [68,151]. ADAMTS protease is a secreted enzyme that acts on various ECM substrates, including substrate proteins and proteoglycans [152]. Chrysin is a natural flavonoid that downregulates collagen I and III expression and improves collagen accumulation. It can inhibit the proliferation of PVSMCs induced by PDGF and intima hyperplasia in PH rats [153].

### 4.6. Right Ventricle Protection

PH affects not only the pulmonary vasculature but also the right ventricle. It has a high morbidity and mortality rate. The effects of PH on the RV range between RV hypertrophy, remodeling, and eventual failure, and are associated with increased mortality. The survival of patients with PH is closely related to RV function. Therefore, protection of RV function is of great significance for the treatment of PH [154,155,156]. Experimental PH treated with resveratrol resulted in significant recovery of RV cardiomyocyte volume, diameter, and contractility, suggesting a protective effect of resveratrol against ventricular dysfunction and pathological remodeling changes in PH [157]. Many reports have shown that *Salvia miltiorrhiza* has a protective effect on the heart. TIIA can relieve ventricular remodeling in rats with pressure overload-induced heart failure by reducing myocardial cell apoptosis [29]. MLB improves right ventricular remodeling in pulmonary hypertension by inhibiting the NOX/VPO1 pathway [44]. In addition, many other natural products, such as hydroxysafflor yellow A, allicin, hawthorn, and breviscapine, have a protective effect on the right ventricle through several pathways [158,159,160,161].

In this section, we have classified and summarized the mechanisms of the natural products mentioned above. The natural products involved in each mechanism are listed. In addition, this information is summarized and improved in the following Table 1.

## 5. Discussion

**Figure 3:**
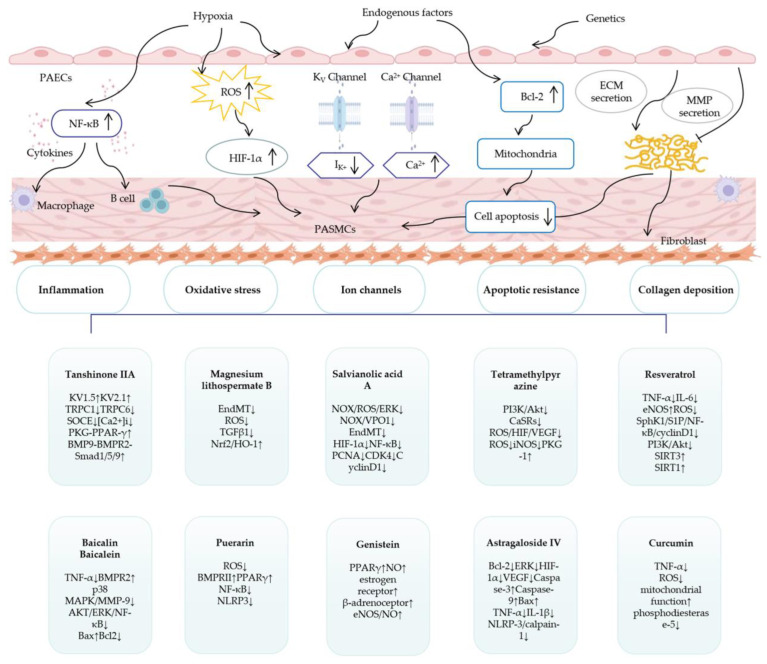
Pulmonary vessels are affected by hypoxia, genetics, and many factors, leading to pulmonary vasoconstriction, vascular remodeling, thrombosis, and right ventricular hypertrophy through inflammation, oxidative stress, apoptotic resistance, ion channel abnormalities, and collagen deposition pathways. A variety of natural products can be used to treat pulmonary hypertension through these mechanisms. Abbreviations: TRPC1, 6: transient receptor potential 1,6; SOCE: store-operated Ca^2+^ entry; PKG-PPAR-γ: hypoxia-inhibited protein kinase G-peroxisome proliferator-activated receptor-γ; BMPR2: bone morphogenetic protein type 2 receptor; BMP9: bone morphogenetic protein 9; EndMT: endothelial-to-mesenchymal transition; ROS: reactive oxygen species; TGFβ1: transforming growth factor-β1; Nrf2/HO-1: NF-E2-related factor 2/heme oxygenase-1; NOX: NADPH oxidases; ERK: extracellular signal-regulated kinase; VPO1: vascular peroxidase-1; HIF-1α: hypoxia-inducible factor-1α; NF-κB: nuclear factor-kappa B; PCNA: proliferating cell nuclear antigen; PI3K/Akt: phosphoinositide-3-kinase/protein kinase B; CaSRs: calcium-sensing receptors; VEGF: vascular endothelial growth factor; iNOS: inducible nitric oxide synthase; PKG-1: cGMP-dependent protein kinases 1; TNF-α: tumor necrosis factor-α; IL-6: interleukin-6; eNOS: endothelial nitric oxide synthase; SphK1/S1P: sphingosine kinase 1/sphingosine-1-phosphate; MAPK: mitogen-activated protein kinase; MMP-9: matrix metalloproteinase-9; Bcl-2: B cell lymphoma-2; Bax: BCL-2-associated X; NLRP3: nucleotide-binding oligomerization domain-like receptor family pyrin domain-containing-3; NO: nitric oxide; IL-1β: interleukin-1β; ECM: extracellular matrix.

PH is a kind of complex, refractory, and poor prognosis multisystem disease that begins with pulmonary vascular disease and ends with right heart failure and death. Its pathological mechanism is complex and has not yet been fully understood. Unfortunately, the current first-line PH therapy is still three kinds of targeted drugs [163]. Small pulmonary vessel proliferation, remodeling, in situ thrombosis, and right ventricular failure have not been well addressed. Therefore, it is urgent to develop new drugs to treat PH from new and effective pathways. In our review, we looked forward to finding breakthroughs in natural products to discover ways to treat PH from more alternative pathways. It is well known that natural products can be used to treat diseases from multiple targets and pathways. Therefore, we reviewed the most popular natural products currently used to treat PH and provided a classified summary of their mechanisms. The natural products involved are described in Section 2. It is found that the natural products derived from *Salvia miltiorrhiza* Bunge are relatively the most studied at present. They exerted their therapeutic effects on PH mainly through modulation of SOCE, PKG-PPAR-γ, BMP9-BMPR2-Smad1/5/9, PI3K/Akt/mTOR, EndMT, Nrf2/HO-1, NOX/OS/ERK, NOX/VPO1, HIF-1, and other pathways [35,36,38,41,42,43,44]. The second is tetramethylpyrazine, which mainly treated PH by regulating CaSRs, ROS/HIF/VEGF, and ROS/iNOS/PKG-1 [51,52,53]. In addition, clinical trials have been conducted on both TIIA and tetramethylpyrazine, which can further prove their therapeutic effects. Next is resveratrol, which regulates Sphk1/S1P/NF-κB/cyclin D1, PI3K/AKT, SIRT3, SIRT1, and other pathways [60,61,62,63]. Then, there are baicalin, baicalein, puerarin, genistein, astragaloside IV, and curcumin, which can be used to treat PH by modulating signaling pathways such as p38 MAPK/MMP-9, Bax/Bcl-2/Cas-3, BMPRII/Smad, PPARγ/PI3K/Akt, eNOS/NO, NLRP-3/calpain-1, and TNF-α [68,74,77,86,90,99]. From the above treatment mechanisms for natural products, we have made a classified summary in Section 3. It was found that most of them play a role in treating PH by influencing inflammatory responses, inhibiting oxidative stress, regulating ion channels, and improving apoptotic resistance and collagen deposition to reduce pulmonary vascular injury, proliferation, and remodeling, and improve right ventricular remodeling. Furthermore, we also summarized the natural products involved in all mechanisms. Many of these natural products, such as TIIA, MLB, resveratrol, ligustrazine, astragaloside IV, baicalin, baicalein, betaine, and allicin, may act by reducing the inflammatory response [38,45,50,60,69,72,90,107,114,115]. MLB, SAA, ligustrazine, resveratrol, 18β-glycyrrhetinic, wogonin, and genistein ameliorate oxidative stress [42,43,129,131,132,134,162]. TIIA, ligustrazine, and resveratrol are also adept at regulating ion channels [33,49,62]. STS, astragaloside IV, salidroside, and apigenin can regulate cell apoptosis [38,89,147,148]. Baicalin and chrysin may reduce collagen deposition [68,153]. Resveratrol, curcumin, hydroxysafflor yellow A, allicin, hawthorn, and other natural products have protective effects on the heart [99,157,158,159,160]. Of course, there are other pathways, such as metabolism, genetics, etc., but they have not been summarized because fewer natural products are involved [65,164]. From the above studies, it can be seen that each natural product can play a role in PH treatment in multiple ways, not the least of which are therapeutic targets that are distinct from traditional targeted drugs. We illustrate this succinctly in Figure 3. We should conduct more in-depth research on these natural products with great development potential, such as TIIA, ligustrazine, resveratrol, puerarin, etc., with a view to exploring new multitargeted, safe, and low-cost drugs for PH treatment.

Natural products have the following advantages in PH treatment: (1) They effectively inhibit pulmonary vascular and RV remodeling by suppressing inflammatory responses, oxidative stress, collagen deposition, and other pathological manifestations. (2) They often have multiple mechanistic pathways to treat PH, which are not achievable with current clinical therapeutics. As mentioned above, puerarin improves hypoxia-induced PVR by reducing autophagy, inhibiting oxidative stress, and activating the BMPRII/Smad and PPARγ/PI3K/Akt signaling pathways [76,77]. However, research on natural products for PH treatment also has some limitations. Some natural plant products for PH treatment have clear mechanisms in experimental studies, but lack effective clinical practice to support their efficacy and cannot accurately guide clinical treatment.

PH is not completely cured, and the disease is usually treated for a long time. However, the three existing targeted drugs have limitations such as high treatment costs and low safety. PH treatment options are currently limited, and natural products have great potential for its treatment. Existing studies mainly elaborated on the therapeutic effect of natural products on PH from the aspects of natural ingredients, fractions, and pure compounds from plants [11,12]. We listed the most important natural products and their therapeutic mechanisms in the treatment of PH, and for the first time, summarized the common mechanisms of natural products, such as inflammation, oxidative stress, and abnormal ion channels, in the treatment of PH from the perspective of the therapeutic mechanisms for PH. In addition, we also identified natural products corresponding to each mechanism and explained the related target pathways. It is expected to provide some new inspiration for future research on PH therapeutic mechanisms and new target drugs and to further evaluate the above PH-related therapeutic mechanisms and natural products or even as tool drugs for research. We look forward to developing more effective medicines for PH treatment to improve patients’ survival rates and quality of life.

## Figures and Tables

**Table 1:** List of some natural products with potential anti-PH effects.

Natural Products	Sources	Model	Dose	mPVP	PVR	RVH	Mechanisms	Phase	Classification
Sodium tanshinone II sulfonate A	*Salvia miltiorrhiza* Bung	HPH rats	10 mg/kg, 30 mg/kg, 3 weeks	+	+	+	IL-6↓IL-8↓TNF-α↓PI3K/AKT/mTOR↓ [38]	Clinical trial Phase 3	Anti-inflammatory
Magnesium lithospermate B	*Salvia miltiorrhiza* Bunge	HPH rats	5, 15 mg/kg, 30 days	+	+	+	EndMT↓HIF-1α↓NF-κB↓MCP-1↓PCNA↓CDK4↓CyclinD1↓ROCK1, 2↓ [45]	Preclinical trials	
Ligustrazine	*Rhizoma Chuanxiong*	MCT-induced PH rats	40, 80, 160 mg/kg, 4 weeks	+	+	+	Inhibit inflammation by regulating the PI3K/AKT [50]	Clinical trial Phase 0	
Resveratrol	Grapes, red wine, peanuts	MCT-induced PH rats	25 mg/kg/, 3 weeks	+	+	+	TNF-α↓IL-1β↓IL-6↓PDGF-α/β↓ [59]	Preclinical trials	
Resveratrol	Grapes, red wine, peanuts	MCT-induced PH rats	25 mg/kg, 4 weeks	+	+	+	SphK1/S1P/NF-κB↓ [60]	Preclinical trials	
Baicalin	*Scutellaria baicalensis* Georgi	MCT-induced PH rats	100 mg/kg, 6 weeks	+	+	+	Regulate the TNF-α/BMPR2 [67]	Preclinical trials	
Baicalin	*Scutellaria baicalensis* Georgi	MCT-induced PH rats	20, 100, 200 mg/kg, 29 days	+	+	+	AKT/ERK/NF-κB↓ [69]	Preclinical trials	
Baicalein	*Scutellaria baicalensis* Georgi	MCT-induced PH rats	50,100 mg/kg, 4 weeks	+	+	+	IL-6↓TNF-α↓IL-1β↓ MAPK↓NF-κB↓ [72]	Preclinical trials	
Baicalein	*Scutellaria baicalensis* Georgi	MCT-induced PH rats	50,100 mg/kg, 4 weeks	+	+	+	NF-κB-BMPR2↓EndMT↓ [73]	Preclinical trials	
Astragaloside IV	*Astragalus membranaceus*	MCT-induced PH rats	10, 30 mg/kg, 3 weeks	+	+	+	TNF-α↓IL-1β↓HIF-1α↓VEGF↓ [89]	Preclinical trials	
Astragaloside IV	*Astragalus membranaceus*	MCT-induced PH rats	40 mg/kg, 80 mg/kg, 4 weeks	+	+	+	NLRP-3/calpain-1↓Caspase-1↓ASC↓IL-18↓IL-1β↓ [90]	Preclinical trials	
Betaine	*Lycium barbarum*	MCT-induced PH rats	100, 200, 400 mg/kg, 6 weeks	+	+	+	MCP-1↓ET-1↓NF-κB↓TNF-α↓IL-1β↓ [107]	Preclinical trials	
Grape seed proanthocyanidin	Grape seeds	MCT-induced PH rats	10 mL/kg, 3 weeks	+	+	+	NF-κB↓IL-1β↓IL-6↓TNF-α↓ [114]	Preclinical trials	
Allicin	*Allium sativum* L.	MCT-induced PH rats	16 mg/kg, 4 weeks	+	+	+	TNF-α↓IL-6↓IL-1β↓CD68↓NFκB p65↓Iκβ↓TGF-β↓α-SMA↓ [115]	Preclinical trials	
Salvianolic acid A	*Salvia miltiorrhiza* Bunge	MCT-induced PH rats	0.3, 1, 3 mg/kg, 4 weeks	+	+	+	Nrf2/HO-1↑ROS↓TGFβ1↓EndMT↓ [42]	Preclinical trials	Oxidative stress
Magnesium lithospermate B	*Salvia miltiorrhiza* Bunge	HPH rats	5, 15 mg/kg, 3 weeks	+	+	+	NOX/ROS/ERK↓NOX2↓NOX4↓ [43]	Preclinical trials	
Tetramethylpyrazine	*Rhizoma Chuanxiong*	MCT-induced PH rats	5 mg/kg, 4 weeks		+	+	ROS/iNOS/PKG↓ [53]	Clinical trial Phase 0	
Resveratrol	Grapes, red wine, peanuts	MCT-induced PH rats	25 mg/kg/, 3 weeks	+	+	+	eNOs↑NOX2↓NOX4↓ [59]	Preclinical trials	
Resveratrol	Grapes, red wine, peanuts	HPH rats	40 mg/kg, 4 weeks	+	+	+	MAPK/ERK1↓PI3K/AKT↓HIF-1 α↓Nrf-2/Trx-1↓ [134]	Preclinical trials	
Trimethoxystilbene	Resveratrol analog	HPH rats	5, 10 mg/kg, 4 weeks	+	+	+	NOX/VPO1↓ [131]	Preclinical trials	
18β-glycyrrhetinic acid	Radix glycyrrhizas	MCT-induced PH rats	25, 50,100 mg/kg, 3 weeks	+	+	+	Nox2↓Nox4↓ [129]	Preclinical trials	
Genistein	Soybeans	CHPH rats	60 mg/kg, 3 weeks	+	+	+	EPO/EPOR↑NO↑ [162]	Preclinical trials	
Tanshinone IIA	*Salvia miltiorrhiza* Bunge	CHPH rats	10 mg/kg, 4 weeks	+	+	+	KV2.1↑KV1.5↑ [33]	Clinical trial Phase 3	Ion channels
Sodium tanshinone IIA sulfonate	*Salvia miltiorrhiza* Bunge	CHPH and MCT-induced PH rats	10 mg/kg, 3 weeks	+	+	+	SOCE↓[Ca^2+^]_i_↓TRPC1↓TRPC6↓ [34]	Clinical trial Phase 3	
Sodium tanshinone IIA sulfonate	Salvia miltiorrhiza Bunge	HPH rats	30 mg/kg, 3 weeks	+	+	+	TRPC↓SOCE↓[Ca^2+^]_i_↓PKG-PPAR-γ↑ [35]	Clinical trial Phase 3	
Sodium tanshinone IIA sulfonate	Salvia milti-orrhiza Bunge	HPH rats	10 mg/kg, 3 weeks	+	+	+	KV2.1↑ [140]	Clinical trial Phase 3	
Tetramethylpyrazine	*Rhizoma Chuanxiong*	HPH, MCT-PH rats	100 mg/kg, 16 weeks		+	+	Inhibition of intracellular calcium homeostasis [49]	Clinical trial Phase 0	
Sodium tanshinone II sulfonate A	*Salvia miltiorrhiza* Bunge	HPH rats	10 mg/kg; 30 mg/kg, 3 weeks	+	+	+	PI3K/AKT/mTOR↓Autophagy↑Bcl-2↓Bax↑ [38]	Clinical trial Phase 3	Apoptotic resistance
Astragaloside IV	*Astragalus membranaceus*	MCT-induced PH rats	10, 30 mg/kg, 3 weeks	+	+	+	Bcl-2↓ERK↓HIF-1α↓VEGF↓Caspase-3↑Caspase-9↑Bax↑ [89]	Preclinical trials	
Apigenin	*Apium graveolens* L. *var. Dulce DC.*	CHPH rats	50, 100 mg/kg, 4 weeks		+	+	Cytochrome C ↑Bax↑Bcl-2↓Caspase-3↑Caspase-9↑HIF-1α-KV1.5↓ [147]	Preclinical trials	
Salidroside	*Rhodiola rosea*	CHPH rats	16, 32, 64 mg/kg, 4 weeks	+	+	+	Bax↑Bcl-2↓Caspase-9↑Cytochrome C↑A2aR↑ [148]	Preclinical trials	
Salidroside	*Rhodiola rosea*	HPH rats	2, 8, 32 mg/kg, 4 weeks		+	+	Regulate the AMPKα1-P53-Bax/Bcl-2-caspase 9-caspase 3 [149]	Preclinical trials	
Baicalin	*Scutellaria baicalensis* Georgi	HPH rats	30 mg/kg, 4 weeks	+	+	+	P38 MAPK/MMP-9↓ [68]	Preclinical trials	Collagen deposition
Baicalin	*Scutellaria baicalensis* Georgi	HPH rats	30 mg/kg, 4 weeks	+	+	+	ADAMTS-1↑Collagen I↓Collagen III↓ [151]	Preclinical trials	
Chrysin	*Oroxylum indicum* (L.) *Vent.* honey, and propolis	HPH rats	50, 100 mg/kg, 4 weeks	+	+	+	Collagen I↓Collagen III↓NOX4↓ [153]	Preclinical trials	
Resveratrol	Grapes, red wine, peanuts	MCT-induced PH rats	20 mg/kg, 6 weeks		+	+	SIRT3↑SERCA↑, prevent right ventricle dysfunction [62]	Preclinical trials	Right ventricle protection
Resveratrol	Grapes, red wine, peanuts	MCT-induced PH rats	20 mg/kg, 6 weeks		+	+	Sirtuin 1↑, improvement of right ventricle and isolated cardiomyocyte [157]	Preclinical trials	
Curcumin	*Curcuma longa* L.	MCT-induced PH rats	50 mg/kg, 4 weeks	+		+	TNF-α↓IL-1β↓Nitrotyrosine↓Fibronectin↓Myosin heavy chain-β↓, attenuate cardiac remodeling [99]	Preclinical trials	
Hydroxysafflor yellow A	*Carthamus tinctorius* L.	HPH rats	25, 50, 75,100 mg/kg, 9 days	+	+	+	PCNA↓Ki67↓, reverse right ventricular hypertrophy [158]	Preclinical trials	

## Data Availability

Not applicable.
